# Supplementary figures and images for: MBL-1/Muscleblind regulates neuronal differentiation and controls the splicing of a terminal selector in Caenorhabditis elegans
Source: PLoS Genet. 2024 Oct 18;20(10):e1011276. doi: 10.1371/journal.pgen.1011276 (PMC11524483; doi:10.1371/journal.pgen.1011276)

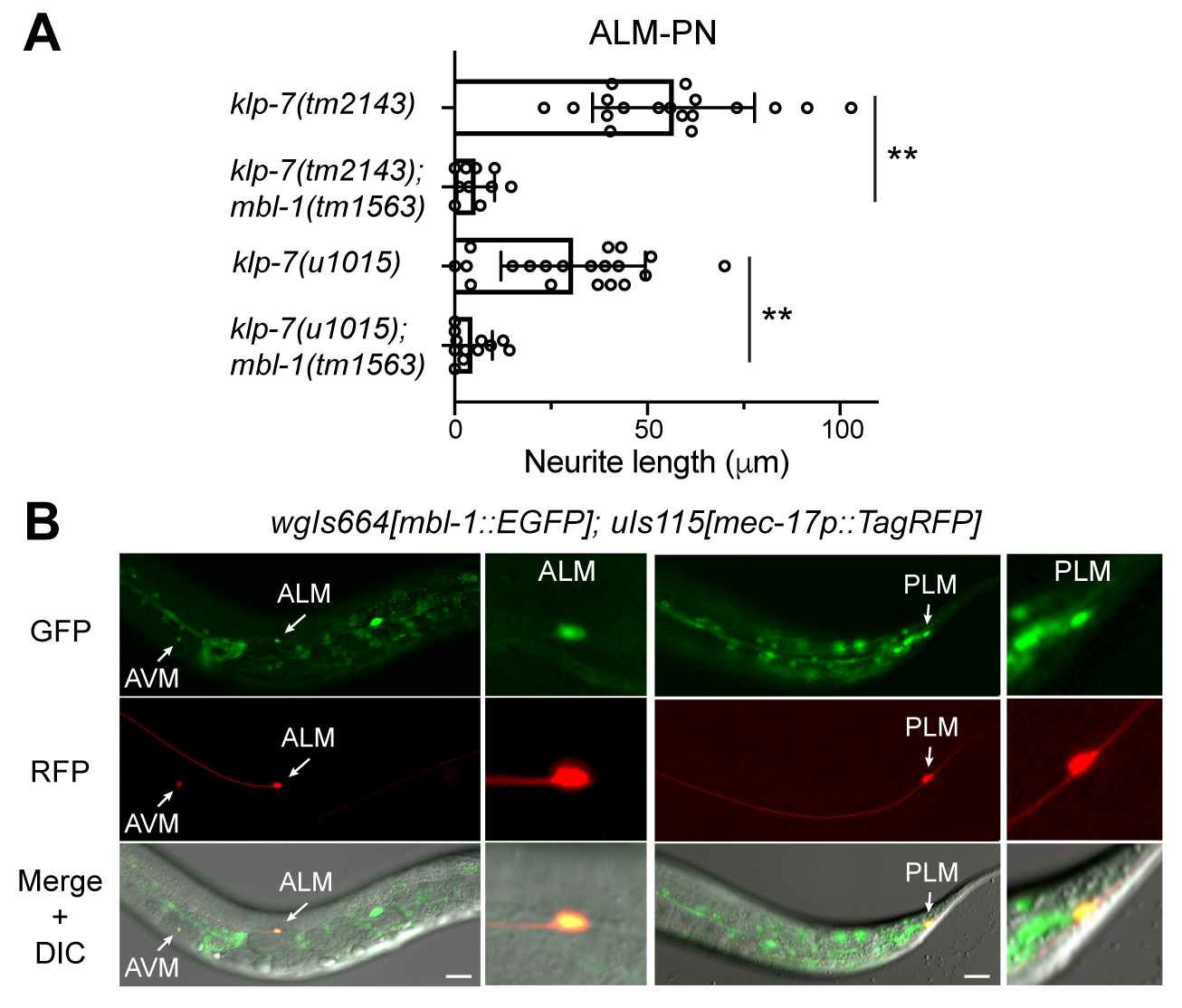

Supplement: S1 Fig — (A) ALM-PN length in the indicated strains. klp-7(tm2143) is a deletion allele, while klp-7(u1015; E95K) is a loss-of-function allele we previously isolated. Double asterisks indicate p < 0.01 in a t-test comparing the indicated two strains. (B) The expression of a fosmid reporter mbl-1::EGFP in the TRNs based on the colocalization with the TRN marker mec-17p::TagRFP. Enlarged images show the ALM and PLM cell bodies. Scale bar = 20 μm. (TIF) [file pgen.1011276.s001.tif]

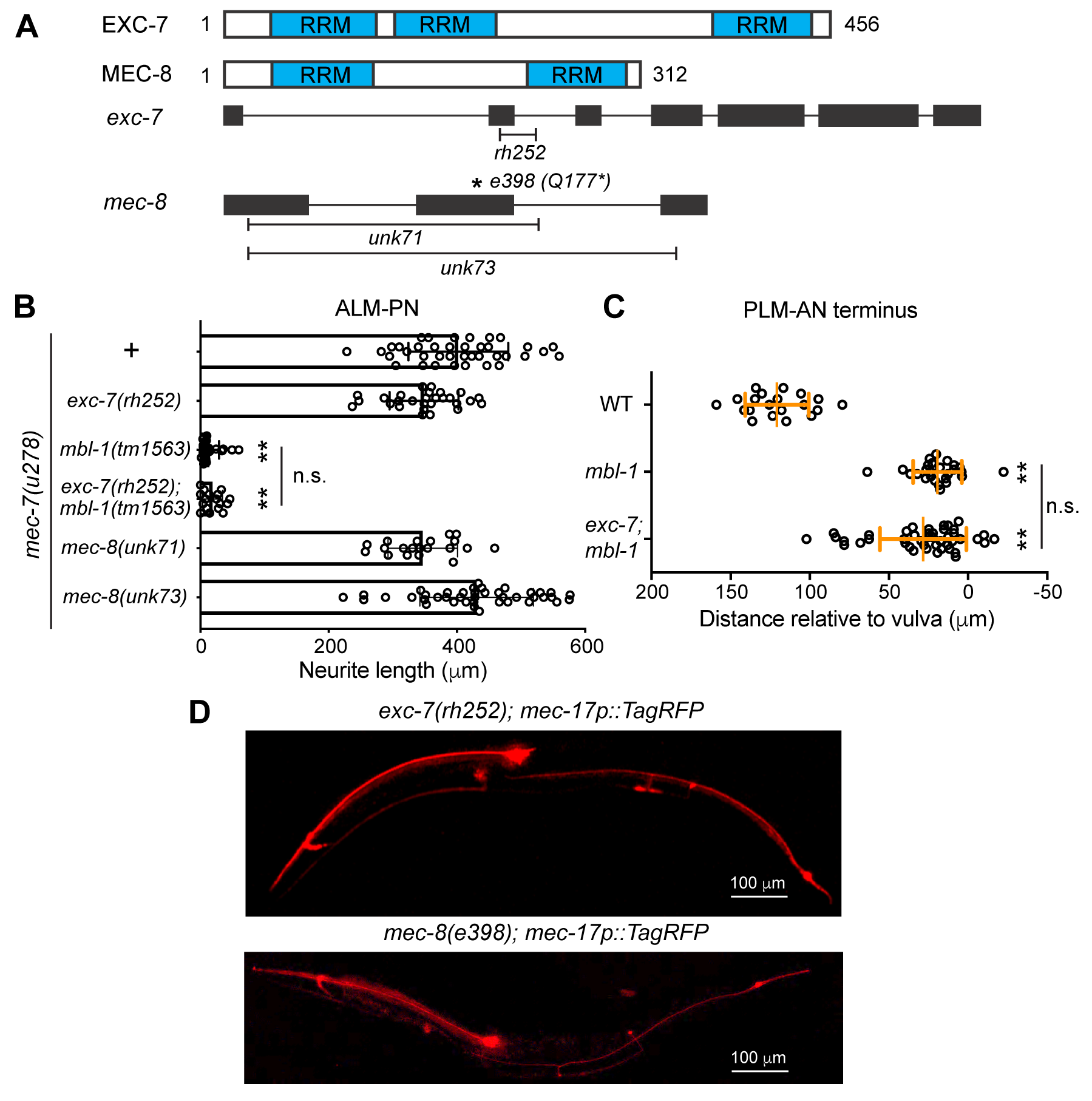

Supplement: S2 Fig — (A) Protein domain structure of EXC-7 and MEC-8; RRM means RNA recognition motif. Gene structure of exc-7 and mec-8 and the molecular changes of their mutations. unk71 and unk73 are deletion alleles generated by CRISPR/Cas9-mediated gene editing in this study. (B) ALM-PN length in mec-7(u278) animals that also carry mutations in various splicing regulators. (C) Quantification of PLM-AN length by the distance from the vulva to the PLM-AN terminus. Positive values mean that PLM-AN grew beyond the vulva, while negative values mean PLM-AN failed to reach the vulva. In (B-C), double asterisks indicate p < 0.01 in a post-ANOVA Tukey’s HSD test. “n.s.” means no statistical significance. (D) Representative images of exc-7 and mec-8 mutants showing normal TRN morphologies. (TIF) [file pgen.1011276.s002.tif]

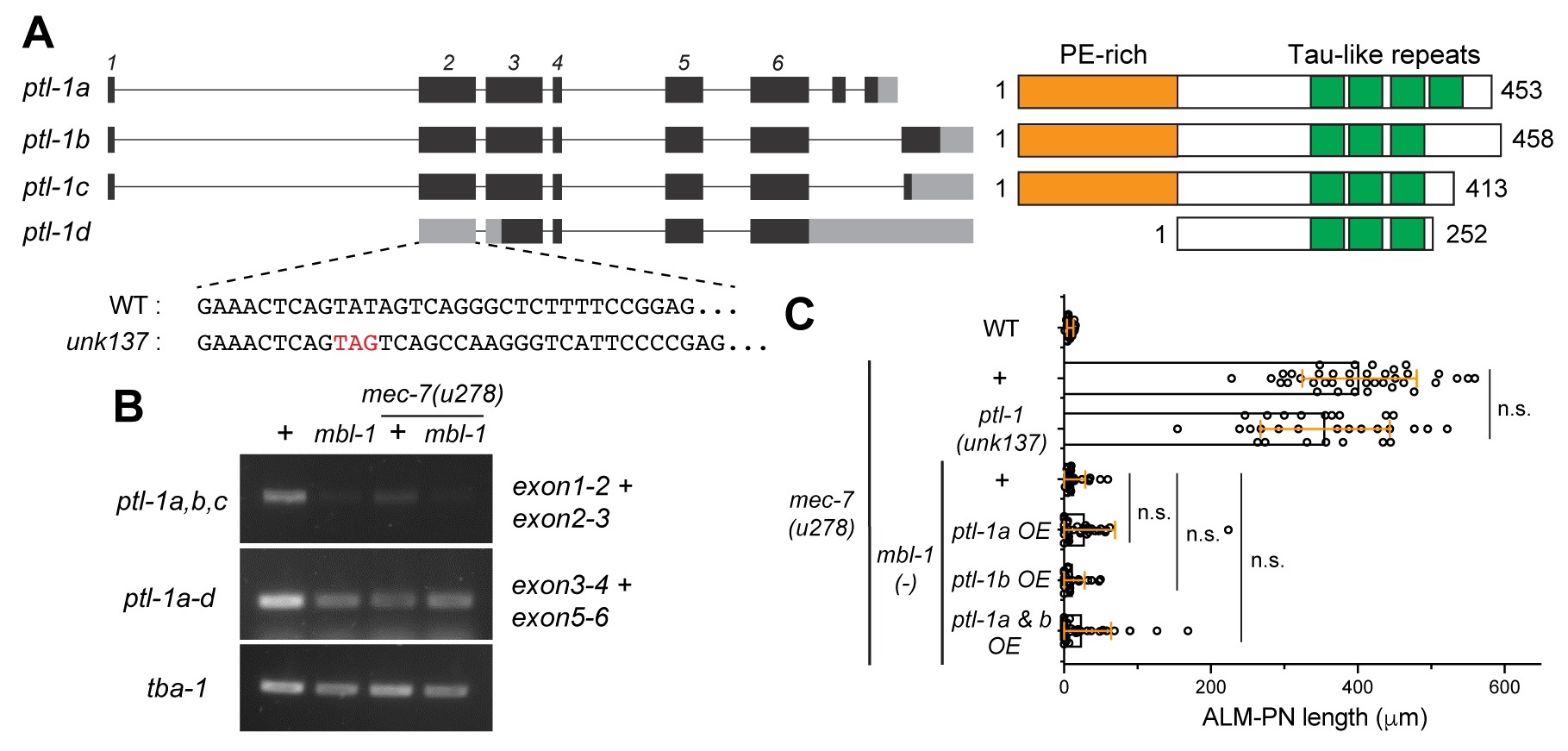

Supplement: S3 Fig — (A) Exon structure of the ptl-1 gene and the domain structures of the PTL-1 protein isoforms. The PE-rich domain is a region that contains many proline-glutamic acid motifs and is mostly coded by exon 1 and exon 2. The unk137 allele (created in this study) has a stop codon (in red) inserted into the exon 2 of the gene. (B) RT-PCR results using isoform-specific primers. To detect the long isoforms (ptl-1a, b, and c), a pair of primers that recognize the exon 1-exon 2 junction and the exon 2–3 junction, respectively, was used. To detect all isoforms, primers that recognize the exon 3–4 junction and the exon 5-exon 6 junction, respectively, were used. (C) ALM-PN lengths of mec-7(u278) animals with a ptl-1(unk137) mutation that inactivated all long isoforms and of mec-7(u278) mbl-1(tm1563) animals with the overexpression of the ptl-1a or ptl-1b cDNAs or both from a TRN-specific mec-17 promoter. “n.s.” means no statistical significance. (TIF) [file pgen.1011276.s003.tif]

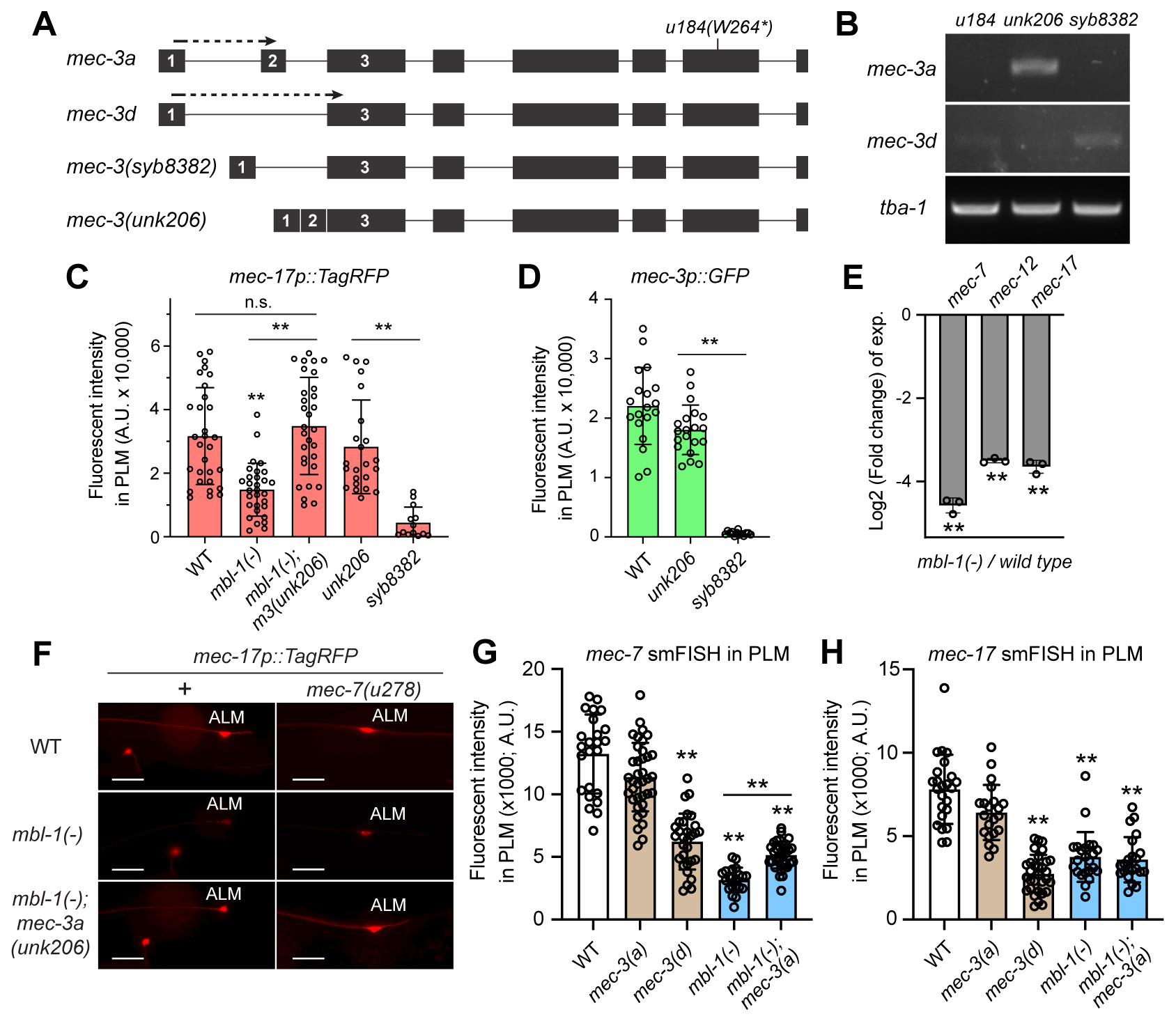

Supplement: S4 Fig — (A) Gene structure of mec-3. The u184 allele serves as a mec-3(-) mutant, while syb8382 and unk206 alleles eliminate the a and d isoforms, respectively. The isoform-specific detection primers used in RT-PCR are indicated by dashed arrows. (B) RT-PCR results of the specific isoform in mec-3 mutants. (C) Fluorescent intensity of the promoter reporter mec-17p::TagRFP in PLM neurons of various strains. For mec-3(syb8382) animals, only the fluorescence of cells that show clear RFP expression were quantified. No statistically significant difference (n.s.) was found between the wild-type and mec-3(unk206) animals. Double asterisks indicate p < 0.01 in a post-ANOVA Tukey’s test. (D) Fluorescent intensity of the promoter reporter mec-3p::GFP in PLM neurons of wild-type, mec-3(unk206), and mec-3(syb8382) animals. For mec-3(syb8382), only the fluorescence of cells that show clear GFP expression were quantified. (E) RT-qPCR results of mec-7, mec-12, and mec-17 mRNA levels in wild-type and mbl-1(tm1563) animals presented as Log2(fold change). (F) Representative images of mec-17p::TagRFP expression in ALM neurons in wild-type, mbl-1(-), mbl-1(-); mec-3(unk206), mec-7(u278), mec-7(u278) mbl-1(-), and mec-7(u278) mbl-1(-); mec-3(unk206) animals. (G-H) smFISH signals against the TRN genes mec-7 and mec-17 in the PLM neurons of various strains. mec-3(a) is the unk206 allele and mec-3(d) is the syb8382 allele. Double asterisks indicate p < 0.01 in Tukey’s HSD tests in comparison with the wild-type animals or between specific pairs. (TIF) [file pgen.1011276.s004.tif]

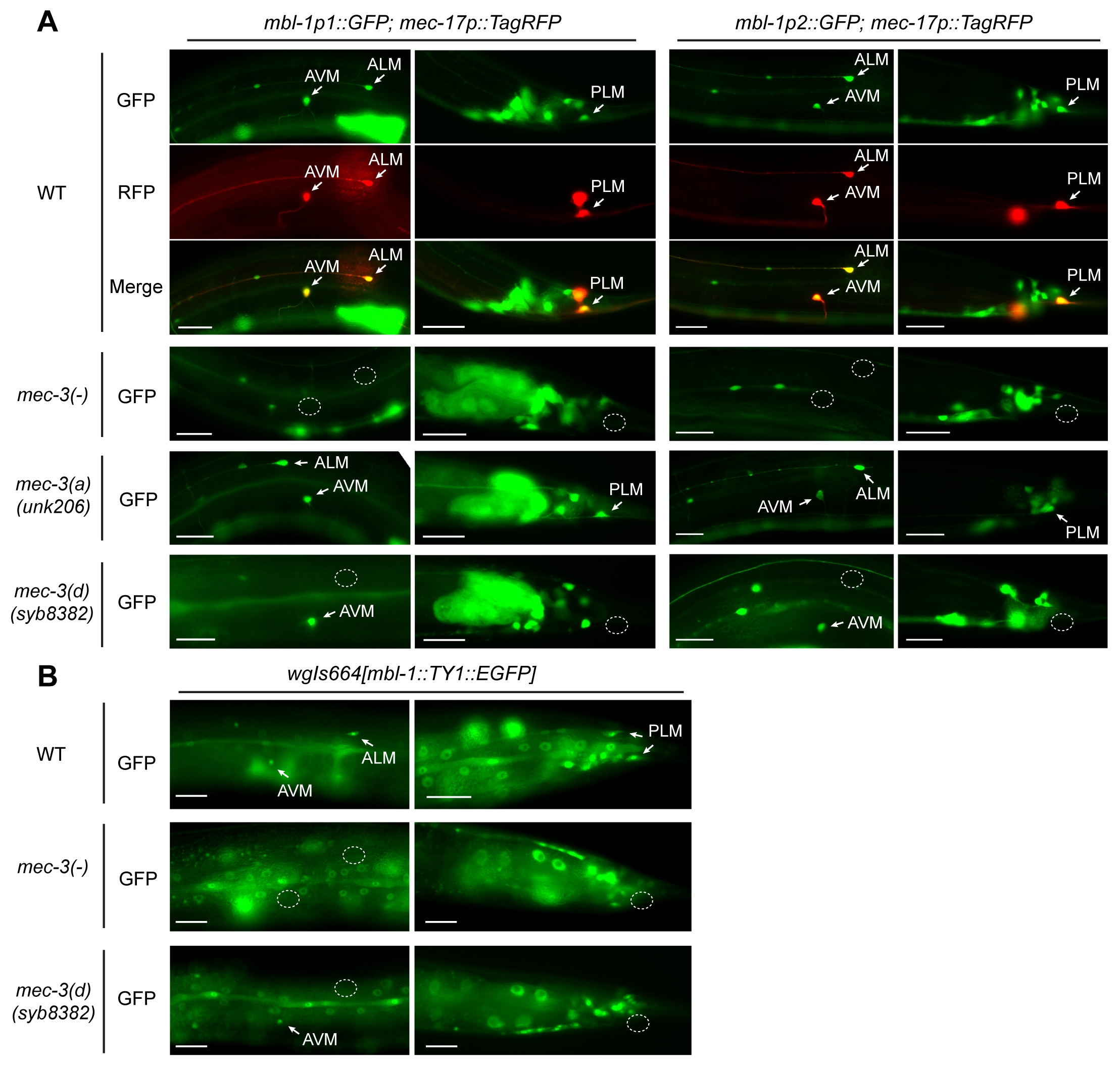

Supplement: S5 Fig — (A) Expression of two promoter reporters for different mbl-1 isoforms, mbl-1p1::GFP and mbl-1p2::GFP (also see Fig 1E), in the ALM, AVM, and PLM neurons. The wild-type and mec-3(unk206) animals showed the TRN expression indicated by the arrows, while the mec-3(u184) and mec-3(syb8382) mutants showed the lack of expression indicated by the dashed circles. Scale bar = 20 μm. (B) The expression of the mbl-1 fosmid reporter wgIs664[mbl-1::TY1::EGFP], which labels all isoforms of mbl-1, was lost in TRNs (dashed circle) in mec-3(u184) and mec-3(syb8382) mutants. (TIF) [file pgen.1011276.s005.tif]

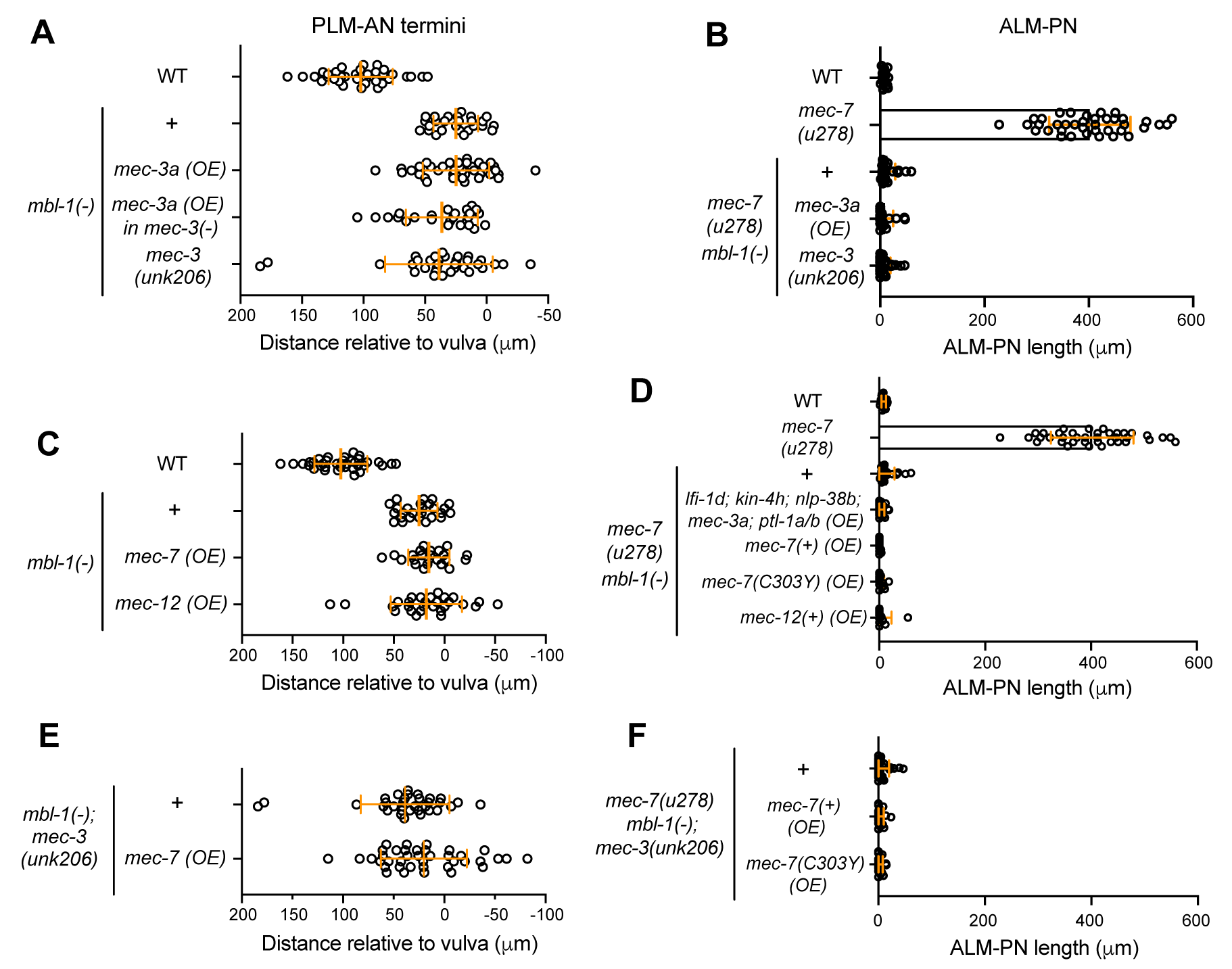

Supplement: S6 Fig — (A) PLM-AN length measured by the distance from the PLM-AN terminus to the vulva in mbl-1(tm1563) mutants with overexpression of mec-3a cDNA or the non-spliceable mec-3(unk206) allele. (B) ALM-PN length in mec-7(u278) mbl-1(-) animals with overexpression of mec-3a cDNA or the mec-3(unk206) allele. (C) PLM-AN length in mbl-1(-) mutants with overexpression of the tubulins mec-7(+) or mec-12(+) from a mec-17 promoter. (D) ALM-PN length in mec-7(u278) mbl-1(-) animals with overexpression of the specific cDNA isoforms of five genes whose splicing were affected by mbl-1(-) mutation, as well as in mec-7(u278) mbl-1(-) animals with the overexpression of mec-7(+) or mec-7(C303Y), which is the mutation found in mec-7(u278), or mec-12(+). (E) PLM-AN length in mbl-1(-); mec-3(unk206) mutants with mec-7(+) overexpression from a mec-17 promoter. (F) ALM-PN length in mec-7(u278) mbl-1(-); mec-3(unk206) mutants with the overexpression of mec-7(+) or mec-7(C303Y). (TIF) [file pgen.1011276.s006.tif]

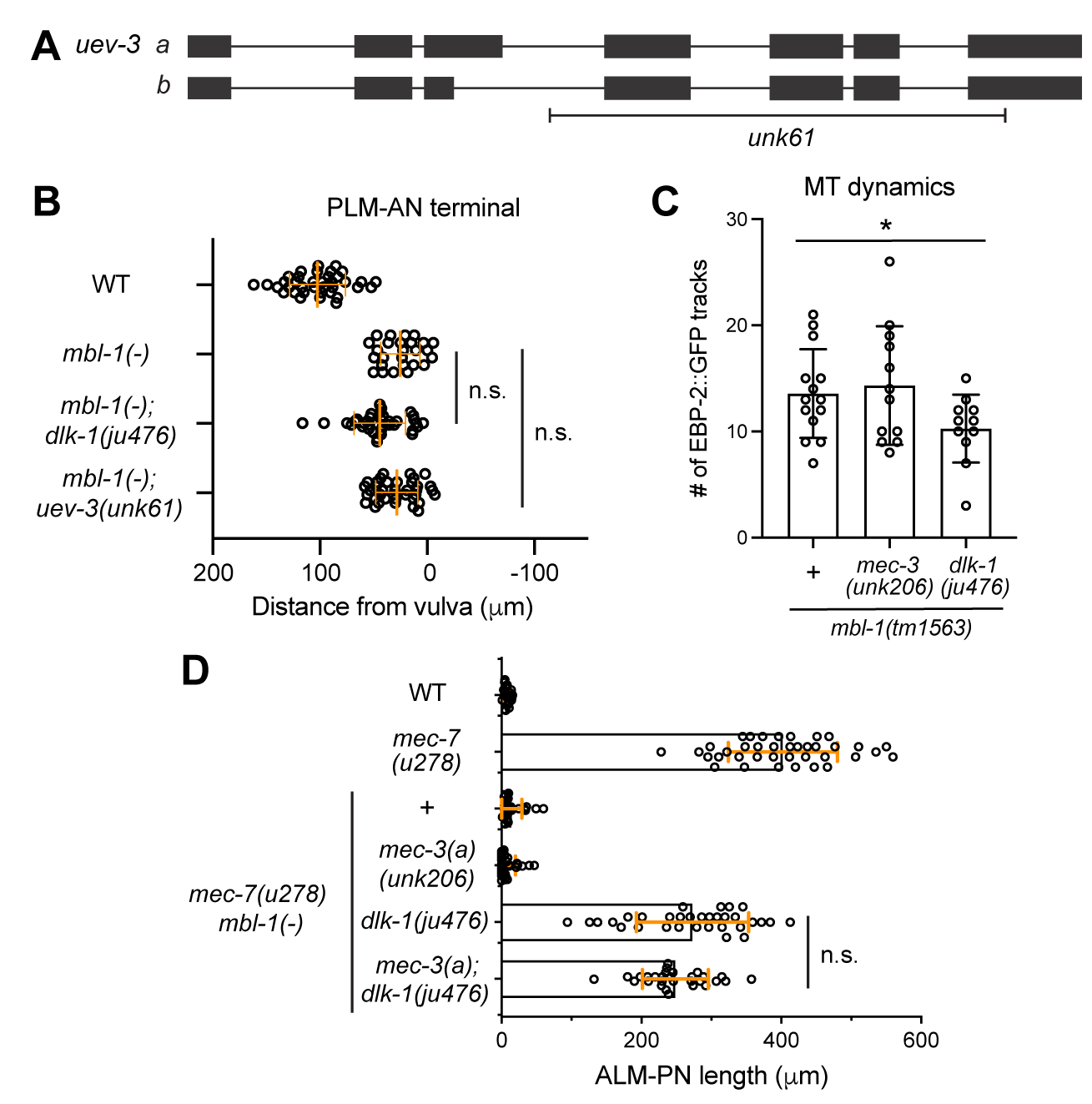

Supplement: S7 Fig — (A) The genomic region that is deleted in the unk61 allele created in this study. (B) PLM-AN length measured by the distance from the PLM-AN terminus to the vulva in mbl-1(tm1563) dlk-1(ju476) and mbl-1(-) uev-3(unk61) double mutants; ju476 is a 5-bp insertion allele that caused frameshift in dlk-1. (C) The number of EBP-2::GFP comets in mbl-1(-) mec-3(unk206) and mbl-1(-) dlk-1(ju476) in a 60 μm region from the PLM cell body within a one-minute recording. Single asterisks indicate p < 0.05 in a post-ANOVA Dunnett’s test. (D) ALM-PN length in mec-7(u278) mbl-1(-); mec-3(unk206) and mec-7(u278) mbl-1(-); dlk-1(ju476) triple mutants and mec-7(u278) mbl-1(-); dlk-1(ju476); mec-3(unk206) quadruple mutants. “n.s.” means no statistical significance in a post-ANOVA Tukey’s test. (TIF) [file pgen.1011276.s007.tif]

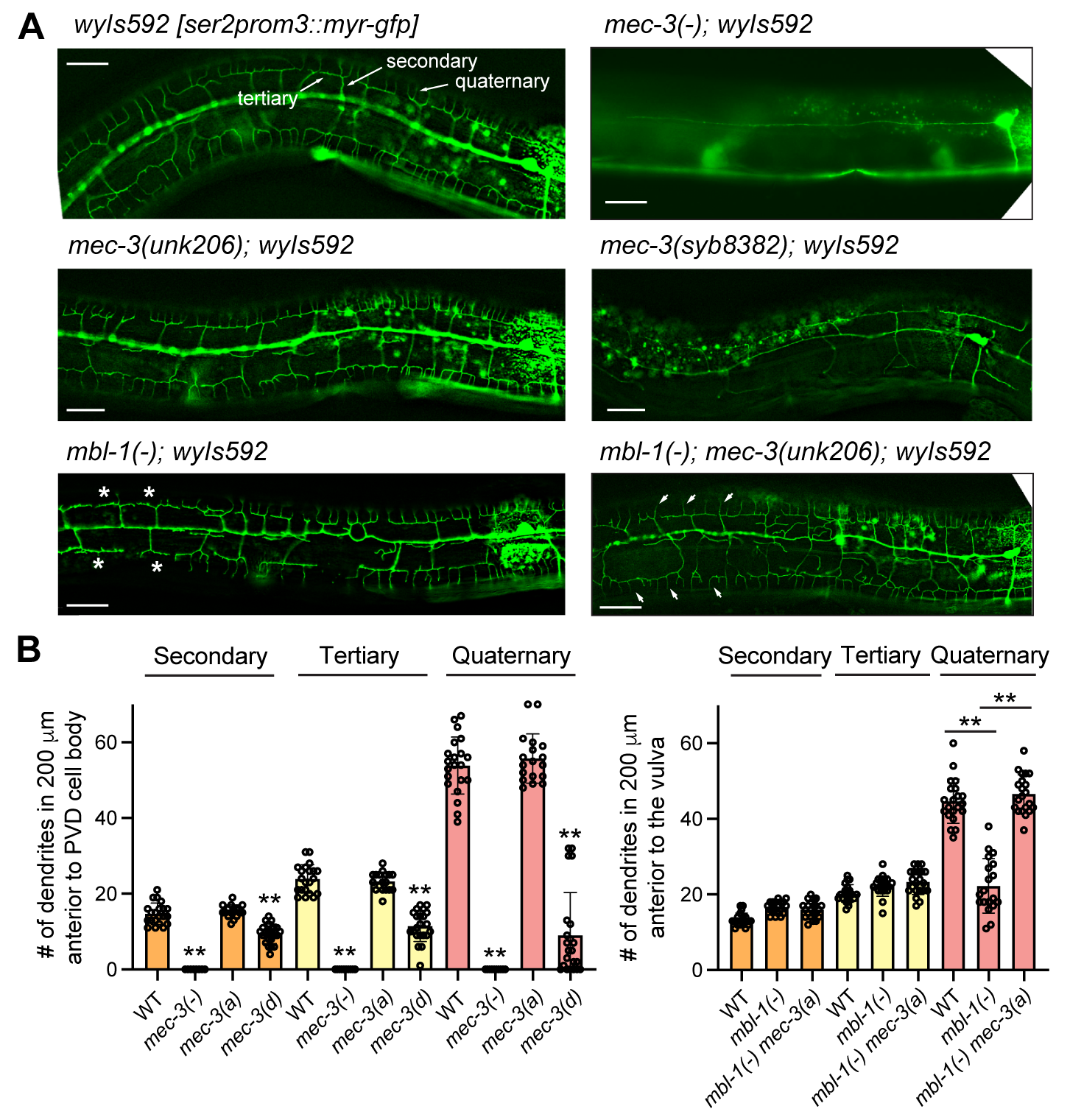

Supplement: S8 Fig — Representative images of PVD morphologies anterior to the cell body in various strains. Arrows in the wild-type image pointed to the secondary, tertiary, and quaternary level dendrites. Asterisks in the mbl-1(-) mutant image indicated the absence of quaternary dendrites on these tertiary dendrites. Arrow heads in the mbl-1(-); mec-3(unk206) mutants indicate the restoration of the quaternary dendrites. (B) Quantification of the number of secondary, tertiary, and quaternary dendrites in a region anterior to the PVD cell body but ≤ 200 μm away from the cell body. mec-3(-), mec-3(a), and mec-3(d) indicate the use of u184, unk206, and syb8382 alleles, respectively. Double asterisks indicate p < 0.01 in Tukey’s HSD tests in comparison with the wild-type animals or between specific pairs. (TIF) [file pgen.1011276.s008.tif]
