# Supplementary material for: MBL-1/Muscleblind regulates neuronal differentiation and controls the splicing of a terminal selector in Caenorhabditis elegans
Source: PLoS Genet. 2024 Oct 18;20(10):e1011276. doi: 10.1371/journal.pgen.1011276 (PMC11524483; doi:10.1371/journal.pgen.1011276)
Supplement: Attachment — Submitted filename: Reviewer Comments and response V3.docx [file pgen.1011276.s016.docx]

**We thank the three reviewers for their constructive comments. Below is a point-by-point response to the reviewers’ comments.**

Reviewer #1: (Comments for the author)

This manuscript by Lee et al. reports a nice and thorough investigation into the molecular consequences of mbl-1 loss on a single neuron type (touch neurons). It presents important points about the relationship between RNA processing and neuronal differentiation/fate. The interpretations are well-founded, and appropriate controls are present. A few changes will make the manuscript suitable for publication:

1. RNA Seq is data is generated but does not appear to be public at this time (on GEO, SRA, or similar databases).

***Response:* The complete RNA sequencing dataset generated in this study has been uploaded to NCBI Gene Expression Omnibus (GEO) with the accession number GSE274472. The dataset is now publicly available at** [**https://www.ncbi.nlm.nih.gov/geo/query/acc.cgi?acc=GSE274472**](https://www.ncbi.nlm.nih.gov/geo/query/acc.cgi?acc=GSE274472)**. We added this information into the revised manuscript.**

2. On page 8, the claim is made that mbl-1(-) causes gene expression changes in mec-12, mec-7, and mec-17. However, the data presented in Figure 4 show that the changes in mec-12 and mec-17 are not significant. Therefore, the claims on page 8 should be removed, as claims about statistically-insignificant gene expression changes should not be reported as gene expression changes.

***Response:* For differential gene expression from the RNA-seq results, all genes listed in Figure 4F had *p* < 0.05 in pairwise comparison between *mec-7(u278) mbl-1(-)* and *mec-7(u278)* or between *mbl-1(-)* and wild-type animals, although some of them (the ones without the double asterisks) showed adjusted *p* values above 0.05 (B-H method was used in DESeq2). Since the *p* value adjustment may lead to false negatives, we listed all the TRN genes that might be downregulated in Figure 4F and stated their *p* value qualifications in the legends.**

**To further confirm this result, we performed additional RT-qPCR to compare the expression of *mec-7*, *mec-12*, and *mec-17* between the wild-type animal and *mbl-1(-)* mutants and found their significant downregulation in *mbl-1(-)* mutants. These new data are now included in Figure S4E (also shown below).**

3. Page 4: a brief sentence explaining what EBP-2::GFP imaging is measuring (e.g. what is EBP-2? Where does it localize?) This would help non-specialists such as myself.

**Response: We added a sentence to explain the experiment of tracking EBP-2::GFP. The sentence is “To monitor MT dynamics, we expressed a GFP-fused MT plus-end binding protein EBP-2 in the TRNs and visualized MT polymerization by tracking the movement of EBP-2::GFP.” (line 149-151).**

Reviewer #2: (Comments for the author)

In this manuscript, Lee and colleagues identified mbl-1 as a genetic suppressor of mec-7(u278) using the ectopic neurite outgrowth as the readout. mbl-1(lf) also suppresses the ectopic neurite formation in klp-7(lf) mutants, which is consistent with the results reported by the Ghosh-Roy group (PMID: 37603562). Since mec-7(u278) causes hyperstable microtubules, the authors examined the MT stability and dynamics and found that mbl-1(lf) animals showed reduced MT stability and increased MT dynamics when compared to the WT. They performed RNAseq and splicing junction analysis and identified many alternative splicing events controlled by MBL-1. Importantly, the splicing of mec-3, a terminal selector critical for the cell fate determination of the TRN and PVD neurons, is dramatically altered by mbl-1(lf). The level of mec-3(a) mRNA is much reduced while that of mec-3(d) is increased in mbl-1(lf). MEC-3(a) shows higher activity to promote the expression of the mec-3 downstream genes, including mec-7, mec-12, and mec-17. Using the CRISPR technology, the authors generated mec-3(syb8382, delta exon 2) and mec-3(unk206, delta intron 1 and intron 2) and showed that correcting the mec-3 alternative splicing defects could partially restore the expression of mec-7 and mec-17. Why is the suppression not 100%? One possibility is that MBL-1 can promote the stability of the mRNAs of the mec-3 target genes, which is reported by the Ghosh-Roy group. The authors further generated a mec-3 splicing reporter and identified three “YGCU(U/G)Y” motifs which can be recognized by MBL-1. Through another round of genetic suppressor screen, they found that the ectopic ALM-PN could form again in mec-7(u278); mbl-1(lf) when dlk-1/pmk-3/cebp-1 is inactivated. Finally, they showed that mbl-1(lf) mutants showed reduced 4o dendrite formation in the PVD neurons, which is largely due to the mec-3 splicing defects (consistent with the findings reported by the Shen lab; PMID: 37729192).

Overall, this study is well-designed and the quality of the data is high. Most of the conclusions are well-supported by the results. The manuscript is well-written and easy to understand. In this independent study, the authors confirmed some of the results reported by the Ghosh-Roy group and the Shen group, and brought some new insights for the mechanisms of how MBL-1 regulates neuronal cell fate determination and morphogenesis. Thus, it deserves to be published in PLoS Genetics. I only have some minor suggestions.

1. In Fig. 2, the authors showed that mbl-1(lf) caused instability of MT. Can this defect be rescued by paclitaxel treatment?

***Response*: Paclitaxel (taxol) treatment had a weak effect in stabilizing MTs in TRNs. We previously showed that hyperstable MTs caused the growth of ectopic ALM-PN, but Paclitaxel only induced a very short ALM-PN in a small percentage of animals (PMID: 33378215; PMID: 23223572). Upon the reviewer’s request, we treated *mbl-1(-)* mutants with paclitaxel and found a weak but not significant rescue of MT instability (panel A below). Being consistent with this result, paclitaxel did not rescue either the PLM-AN defects of *mbl-1(-)* mutants (panel B) or the ALM-PN growth in the *mec-7(u278); mbl-1(-)* mutants (panel C). Thus, we concluded that the effects of paclitaxel may not be strong enough to fully rescue the defects in *mbl-1(-)* mutants.**

**Moreover, given that paclitaxel stabilizes MTs by binding to the β-tubulin submit, if there is a general reduction in tubulin (e.g., *mec-7* and *mec-12*) expression levels in *mbl-1(-)* mutants, we may not expect paclitaxel to work effectively to rescue the MT instability in *mbl-1(-)* mutants.**

2. Any explanation why there are more mitochondria in the proximal part of PLM-AN? It seems to me that the number of mitochondria in the entire PLM-AN is increased in mbl-1(lf) when compared to the WT. Is that true?

***Response*: We do not have a clear answer for the seemingly increased mitochondria number in the proximal part of PLM-AN. We hypothesize that increased MT dynamics may lead to more frequent MT depolymerization and polymerization and the generation of many short MTs proximal to the cell body, which may lead to an increased MT-mediated mitochondrial transport and thus overaccumulation of mitochondria in the proximal axon. Although the total number of mitochondria in the *mbl-1(-)* axon appears to be higher than the wild-type animals, we observed bigger variation in the distal segment in *mbl-1(-)* mutants compared to the wild-type animals.**

3. I am curious whether the defects of MT stability and polarity in mbl-1(lf) can be suppressed by mec-3(unk206) and dlk-1(lf).

***Response*: We performed additional experiment to track EBP-2 comets in *mbl-1(-) mec-3(unk206)* and *mbl-1(-) dlk-1(-)* animals. We found that the *mec-3(unk206)* mutation did not rescue the MT stability and polarity in *mbl-1(-)* mutants, which is consistent with the fact that *mec-3(unk206)* did not rescue the neurite growth defects in *mbl-1(-)* mutants. On the other hand, the *dlk-1(-)* mutation partially suppressed the MT instability in *mbl-1(-)* mutants, although it did not suppress the MT polarity defects. Some of the data are included in Figure S7 and shown below for easy reference.**

4. It would be valuable to compare the transcriptomes of mbl-1(lf) and mbl-1(lf); mec-3(unk206) and analyze the splicing junctions.

***Response:* We did not perform this analysis because the main goal of this study is to identify MBL-1 splicing targets that may be involved in regulating neuronal differentiation. Through two sets of transcriptomic comparison [wild type vs *mbl-1(-)* and *mec-7* vs *mec-7; mbl-1(-)*], we identified several genes whose splicing is regulated by *mbl-1* (Figure 3). Although *mec-3* is among the MBL-1 splicing targets, we do not expect MBL-1 regulates other targets through *mec-3*, which codes for a transcription factor. Moreover, we have tested all the MBL-1 targets that showed expression in the TRNs and found that *mec-3* is the only gene that may serve as a functional effector downstream of MBL-1. Thus, even if MBL-1 regulates the splicing of other genes through *mec-3*, these regulations may not show clear phenotypes in TRN differentiation.**

5. For Fig. 4B, please clearly indicate that the experiments were performed in the mec-3(lf) genetic background in this panel (although this information can be found in the figure legend). And explain why there is no much difference in the PLM neurons for the two reporters?

**Response: We added a notation in Figure 4B to indicate that the experiment was done in *mec-3(-)* mutants.**

**The fact that animals with *mec-3(d)* overexpression showed TRN marker expression in PLM but no other TRN subtypes suggests that PLM neurons may have a lower threshold for TRN fate activation. Given that MEC-3d still has some residual activities, we expect that MEC-3d can still drive the expression of downstream genes, although not as efficient as MEC-3a. PLM appears to require less MEC-3 activity to activate the TRN genes compared to other TRN subtypes, as we can see that *mec-3(a)* overexpression also induced TRN marker expression at a higher percentage in PLM neurons compared to other TRN subtypes.**

**Moreover, although the number of PLM neurons expressing the TRN markers is similar between the *mec-3(a)* and *mec-3(d)* overexpression strains, their expression levels are quite different. PLMs with *mec-3(d)* tend to have lower GFP and RFP expression level compared to *mec-3(a)*, confirming that MEC-3a is better at activating downstream genes than MEC-3d. The same goes for the strains carrying the *mec-3(a)* only (*unk206*) or *mec-3(d)* only (*syb8382*) alleles. We added the quantification of GFP and RFP intensity in PLM neurons of those strains in Figure S4C and S4D (also pasted below).**

6. For Fig. 4C, also explain why the expression of mec-3p::gfp is reduced in mec-3(d) mutants, while that of mec-17p::tagRFP is not? How many animals were quantified for Fig. 4B and 4C? The information of the sample size should be indicated in the figure/figure legend/somewhere else.

***Response*: For Figure 4C, we were also surprised that *mec-3p::GFP* showed different expression pattern from *mec-17p::TagRFP* in the *mec-3(d)* only allele. We suspect that the activation of *mec-3* promoter may have a higher threshold in PLM neurons compared to *mec-17* promoter. In the wild-type animals, *mec-17* (161258.8 TPM according to scRNA-seq data) is expressed at a much higher level than *mec-3* (2416.921 TPM), suggesting that *mec-17* promoter may be easier to activate than *mec-3* promoter. So, it is possible that the MEC-3d expressed from the endogenous locus is sufficient to activate *mec-17* promoter but not the *mec-3* promoter. Nevertheless, as shown above, the expression level of *mec-17p::TagRFP* was much lower in *mec-3(d)*-only animals compared to *mec-3(a)*-only animals.**

**The number of animals quantified for Figure 4B and 4C are added to the Figure legends in the revised manuscript. Over 45 animals were counted for each strain.**

7. For Fig. 4F, it’s better to indicate the results as: mec-7(u278); mbl-1(-)/mec-7(u278); and mbl-1(-)/WT on top of the panels.

***Response*: Corrected.**

8. For Fig. 4I, any significant difference between mbl-1(-) and mbl-1(-); mec-3(a)? Even if it is NS, it should be indicated.

***Response*: We added notations for “not significant” in pairwise comparison.**

9. For Fig. 5, the site 1 is clearly in the exon 2. Why did the authors claim that all the three sites are downstream of the included exon?

**Response: We believe the reviewer is referring to the sentence “MBL-1 promotes *mec-3* splicing through three “YGCU(U/G)Y” motifs located downstream of the included exon …” in the Abstract. We have changed it to “MBL-1 promotes *mec-3* splicing through three “YGCU(U/G)Y” motifs located in or downstream of the included exon”.**

10. For Fig. 6, since mbl-1(lf) alone shows PLM-AN outgrowth defect, did the authors ever examine whether loss of dlk-1 and the downstream genes can suppress the above-mentioned defect?

**Response: During the revision, we created *mbl-1(-); dlk-1(-)* and *mbl-1(-); uev-3(-)* double mutants but did not observe significant rescue of the PLM-AN growth defect (Figure S7B; also shown below). We reason that since the inactivation of the DLK-1 pathway only partially restored ALM-PN in the *mec-7(u278)* background, the rescuing effect is limited. Given that the PLM-AN defect in *mbl-1(-)* mutants is mild and may be less sensitive than the ectopic ALM-PN growth, mutations in the DLK-1 pathway is not able to rescue the PLM-AN outgrowth defect. This finding also points out the importance of having a sensitized background for genetic screens. Nevertheless, when tracking the EBP-2::GFP comets, we can still observe reduced MT dynamics in *mbl-1(-); dlk-1(-)* double mutants compared to the *mbl-1(-)* single mutants, suggesting that the loss of DLK-1 may still suppress the defects in *mbl-1(-)* mutants to some extent.**

11. The authors proposed that MBL-1 functions upstream of the DLK-1 pathway. The only result supporting this conclusion is the genetic suppression. I am not totally convinced as we may still see the suppression if they act in redundant genetic pathways. The ALM-PN outgrowth phenotype is just the readout of the overall neurite outgrowth activity of the ALM neuron.

**Response: We agree with the reviewer. In the revised manuscript, we added one sentence stating “Nevertheless, we could not rule out the possibility that the MAPK pathway acts in parallel to antagonize the activity of MBL-1 in promoting neurite growth.” (line 440-442).**

12. For Fig. 7A, it is better if the order of the images is consistent with Fig. 7B.

**Response: We thank the reviewer for pointing out the inconsistency. The order of the images is now adjusted for both Figure 7 and Figure S8.**

13. For Fig. S1, it is nice to see the results using the mbl-1 fosmid-based reporter. In Fig. S5, the authors analyzed the two mbl-1 promoter-gfp fusion reporters and concluded that mec-3 positively regulates the transcription of mbl-1. Is the MBL-1 protein expression level affected by loss of mec-3? This can be done using the fosmid-based reporter.

***Response*: We thank the reviewer for the suggestion. In the revised manuscript, we examined the *mbl-1::EGFP* fosmid reporter and found that the MBL-1 protein is similarly lost in the TRNs in *mec-3(-)* mutants and the *mec-3(d)* only mutants (see Figure S5B; also shown below).**

14. The authors stated: the mec-3a was required for the expression of likely all isoforms of mbl-1 and mec-3d alone was not sufficient to activate mbl-1. The two transcriptional reporters do not report the transcription of the isoform d and e. Thus, it is not proper to say “all isoforms”.

***Response*: Since we added the results that the expression of fosmid-based *mbl-1::EGFP* reporter, which labels all *mbl-1* isoforms, is also lost in *mec-3(-)* and *mec-3(d)* only mutants. It becomes reasonable to state that “*mec-3a* was required for the expression of likely all isoforms of *mbl-1*” in the revised manuscript.**

15. For Fig. S8B, the dendrite formation phenotypes are quantified in different regions for different genotypes, which is confusing. As mbl-1(lf) mutants show reduced branching in the distal part, but not the proximal part, of the PVD neurons, it is better to show the number of dendritic branches in the 200 um region anterior to the vulva for all the strains. mec-3(d) was incorrectly labeled as mec-3(b). m3a should be labeled as mec-3(a).

***Response*: For the PVD morphogenesis, we quantified secondary, tertiary, and quaternary dendrites in regions both anterior (Figure S8) and posterior (Figure 7) to PVD cell body to maximally capture the phenotypic difference among strains. In Figure S8, we showed the number of dendrites in the 200 μm region anterior to the vulva for *mbl-1(-)* mutants and *mbl-1(-) mec-3(a)* mutants only, because it is very clear that *mec-3(-)* and *mec-3(d)* only mutants had almost no secondary and above dendrites in this distal segment (Figure S8B). We do not feel that our data presentation is confusing to the readers.**

**We thank the reviewer for pointing out the labeling errors, which are corrected in the revised manuscript.**

16. For Table S6, the authors should list all the strains used in this study in the order of the figures, and clearly indicate the figures in which the strains are used. At least some of the strains used in Figure 7 are not included in the current version.

***Response*: In the revised manuscript, we have provided an updated list of strains used in this study in Table S6.**

Reviewer #3: (Comments for the author)

In this manuscript, a genetic screen by Lee et al. unravels the role of the splicing regulator mbl-1 in promoting neurite growth, consistent with the findings of Puri et al. (2023). The authors of this manuscript also demonstrate the role of mbl-1 in regulating the splicing of mec-3, the terminal selector for the touch receptor neuron (TRN) fate, which supports the findings of Xie et al. (2023). Overall, this manuscript is a valuable contribution to existing literature and the authors have done well to support their claims with solid data. In particular, the experiments demonstrating the role of mbl-1 in regulating mec-3 splicing are elegant and compelling.

The authors begin the manuscript with a genetic screen to reveal neurite outgrowth regulators, in which they find mbl-1. They demonstrate convincingly that mbl-1 regulates neurite outgrowth. In attempting to find targets of mbl-1, they perform RNA-seq, in which they discover that mbl-1 regulates the splicing of seven TRN-expressed genes. Among these genes, they focus on mec-3 because it is the only gene in which, upon overexpression of the mec-3d isoform upregulated in mbl-1(-), they find a suppression of neurite outgrowth which is a similar phenotype to mbl-1(-), albeit milder. They then proceed to present compelling evidence that mbl-1 regulates mec-3 splicing. Overall, the authors did well to support their claims that (1) mbl-1 regulates neurite outgrowth and (2) mbl-1 regulates mec-3 splicing.

Major comments:

1. In Fig. 3C. Since much of the data contained in the paper rest on the differential isoform usage of mec-3 in mbl-1 mutant animals, we suggest performing a qPCR to measure the expression levels of mec-3a and mec-3d in WT, mbl-1, mec-7 and mec-7;mbl-1 mutants (See as example Fig6F in this manuscript and Fig6C in Puri et al.). This would corroborate the RT-PCR data. In addition, the authors could also measure the overall levels of mec-3 in all the conditions mentioned above using qPCR.

**Response: As the reviewer suggested, we have performed additional RT-qPCR experiment to confirm the downregulation of *mec-3a* and upregulation of *mec-3d* levels in *mbl-1(-)* mutants compared to the wild type and in the *mec-7(u278) mbl-1(-)* mutants compared to the *mec-7(u278)* single mutants. We also designed qPCR primers that bind to all *mec-3* isoforms and found that the overall level of *mec-3* is slightly increased in *mbl-1(-)* mutants compared to the wild-type and in *mec-7(u278) mbl-1(-)* mutants compared to *mec-7(u278)* likely due to the upregulation of *mec-3d*. The results are shown in Figure 3D and shown below for easy reference.**

2. While less critical, this experiment could help understand whether mbl-1 is specifically controlling splicing of mec-3 or also its overall levels. The authors also need to show quantification and statistical analysis while comparing RT-PCR band intensities in Fig 3C.

**Response: We have repeated the RT-PCR experiment a few times and measured the band intensity. The quantification supported the downregulation of *mec-3a* isoform and upregulation of *mec-3d* isoform upon the loss of *mbl-1* in both wild-type and *mec-7(u278)* background. We also used primers that bind to all *mec-3* isoforms and confirmed that the overall level of *mec-3* is slightly increased in *mbl-1(-)* mutants. The results are shown in the new Figure 3C and pasted below for easy reference.**

3. In mbl-1(-), the expression of terminal identity genes such as mec-7 and mec-17 is reduced (Fig4G, 4H, 4I). This could be due to the aberrant expression of mec-3d in mbl-1 mutants, but the authors show removing mec-3d is not sufficient to restore control levels of mec-7 and mec-17 (Fig 4h and 4I). The authors suggest mbl-1 could target both mec-3 (as shown in this manuscript) and mec-7 (as shown by Puri et al.). To corroborate this interesting model the authors should test if they can rescue the ALM and PLM neurite phenotype in an mbl-1 background where the mec-3d isoform has been removed (mec-3(A)) and mec-7 expression has been restored. The ideal experiment would be to find if the same YGCU(U/G)Y motif is also present in mec-7 and mutate it so as to abrogate the possible direct regulation between mbl-1 and mec-7. This approach was very successful for the authors in this manuscript (See Fig5). If this is not feasible then the authors should try again to over-express mec-7 in a mbl-1;mec-3(a) background to test if this is sufficient to fully rescue the ALM-PN phenotype (e.g mec-7(u278) vs mbl-1;mec-3(a);mec-17p:mec-7 or the PLM-AN phenotype.

***Response*: In our hands, overexpression of tubulin genes (e.g., *mec-7*) did not rescue the neurite growth defects in *mbl-1(-)* mutants (Figure S6C and S6D).**

**Based on the reviewer’s suggestion, we performed additional experiment to overexpress *mec-7* in *mbl-1; mec-3(unk206)* double mutants and still could not rescue the PLM-AN defects. We also overexpressed either *mec-7(+)* or *mec-7(C303Y)* in *mec-7(u278)* *mbl-1; mec-3(unk206)* triple mutants and did not observe any rescue of ALM-PN growth (Figure S6E and S6F; also shown below). Thus, we conclude that even after correcting *mec-3* splicing problems, restoring the expression of tubulins alone may not be sufficient to rescue the neurite growth defects in *mbl-1(-)* mutants. There are likely additional downstream effectors that need to be corrected.**

4. The authors nicely show that overexpressing both mec-3d and dlk-1a has an additive effect in suppressing mec-7(u278) extended ALM-PN neurite. They should also test if, in an mec-7(u278);mbl-1 background, removing mec-3d isoform (using mec-3(a) allele) together with removing dlk-1 (or any other member of the MAPK pathway) has a stronger effect than removing dlk-1 alone. This should be done by testing (as an example) the ALM-PN neurite’s length in mec-7(u278);mbl-1 vs mec-7(u278);mbl-1;mec-3(d) vs mec-7(u278);mbl-1;dlk-1 vs mec-7(u278);mbl-1;mec-3(d);dlk-1.

**Response: During the revision of the manuscript, we performed the experiment the reviewer suggested by crossing *mec-3(unk206)* and *dlk-1(-)* together in the *mec-7(u278) mbl-1(-)* mutant background. However, unlike the additive effects between the overexpression of *mec-3d* and *dlk-1a*, we did not observe additive effects between *mec-3(a)* and *dlk-1(-)*. ALM-PN was not rescued at all in *mec-7(u278) mbl-1(-); mec-3(a)* and the partial rescue in *mec-7(u278) mbl-1(-); dlk-1(-)* was not enhanced in the *mec-7(u278) mbl-1(-); dlk-1(-); mec-3(a)* quadruple mutants (Figure S7D; also shown below). We reason that there are other splicing or RNA stability defects in *mbl-1(-)* mutants that need to be corrected to fully rescue the ALM-PN defects.**

Minor comments:

1. Please indicate the allele for mec-17p::TagRFP reporter in Fig. 4D. In the methods section, two mec-17p::TagRFP reporter alleles are mentioned, uIs115 and uIs134, but only uIs115 is cited in the main text.

***Response*: The two reporters are basically the same but are located on two chromosomes. We mostly used *uIs115*, which is on Chromosome IV, but in some experiments the gene of interests (e.g., *mec-3*) is also located on Chr IV. In those cases, we had to switch to *uIs134*. The specific use of the reporters can be found in the strain list in Table S6.**

2. The authors should provide more details about their RNAseq experiments. It would be useful to show some standard quality control plots like a PCA plot with all the samples.

***Response*: The analysis of the RNA-seq results was carried out using standard software (STAR and DESeq2). The number of reads and mapped reads are similar across samples (listed below).**

**We also conducted principal component analysis (PCA), and the top three principal components are listed below. PC1 counted for >88% of the variance. At the transcriptome level, the samples do not separate well, which is consistent with the fact that very few (10~20) genes showed statistically significant difference in gene expression between the wild type and *mbl-1(-)* mutants. One should note that these are whole-organism RNA-seq analysis; if we could do TRN-specific RNA-seq or single-cell RNA-seq we may find more differentially expressed genes.**

**The raw sequencing data is now publicly available at NCBI Gene Expression Omnibus (GEO) with the accession number GSE274472.**

3. The authors should address the fact that mec-7(u278) seems to have an effect on the expression of several genes that are also regulated by mbl-1 (for example WT vs mec-7(u278) on 1) mec-3a and mec-3d(perhaps) in Fig3C 2) ptl-1a,b,c and ptl-1a-d in FigS3 3) mec-17p:TagRFP in Fig4G. A possible regulation of these genes by changes in mec-7 could further complicate the model the authors are presenting. Considering that mbl-1 affect the expression of mec-7 this possibility should be at least addressed.

***Response*: It is known that changes in microtubule polymerization dynamics would affect gene expression in the touch receptor neurons (PMID: 21368137). We have also previously reported the reduced expression of *mec-17p::TagRFP* reporter in *mec-7(u278)* mutants (Zheng et al., 2017, MBoC; PMID: 28835377). Similar downregulation of TRN markers was found with many tubulin missense mutations (Zheng et al., 2017, MBoC; PMID: 28835377). So, it is expected that *mec-7(u278)* may generally affect gene expression in TRNs. This is why we performed experiments in both the presence and absence of the *mec-7(u278)* mutant background and found consistent results that MBL-1 positively regulates *mec-17* promoter activity (Figure 4G). In Figure 4H and I, we further confirmed that MBL-1 regulates endogenous *mec-7* and *mec-17* expression in the wild-type background without *mec-7(u278)*. So, we think this MBL-1-mediated regulation is independent of the potential complication from using *mec-7(u278)* allele. Nevertheless, we added a sentence to explain the reduced expression of *mec-17p:TagRFP* reporter in the *mec-7(u278)* mutants compared to the wild type in the revised manuscript.**

4. Puri et al. (2023) shows that the mbl-1(-) allele tm1563 reduces anterior and posterior touch sensitivity. The author’s data in Fig 4J for anterior touch is similar to Puri et al., although they used a touch response index to measure sensitivity (versus number of responses for this manuscript). However, while Puri et al. sees an effect for posterior touch, this manuscript does not. The authors should mention this in this manuscript.

***Response*: In our hands, we could only observe slight reduction in the anterior touch response in the *mbl-1(-)* mutants and no statistically significant changes in the posterior touch response. This finding is consistent with the result that PLM differentiation requires a lower threshold of MEC-3 activity compared to ALM neurons, as MEC-3d alone appears to be sufficient to drive TRN marker expression in PLM neurons (Figure 4B and 4C). We mentioned this discrepancy between our results and Puri et al.’s results in the revised manuscript.**

5. Authors mention that skipping of exon 2 by shorter mec-3 isoforms leads to truncation of the LIM domain. Looking at the genome browser in Wormbase however, it does not seem that exon 2 contributes to the LIM domain. In Fig4A, the authors should put the schematic of LIM domain motifs directly below the schematic of the exon structure of mec-3 to clarify the correspondence between the two.

***Response*: We prefer to keep the current presentation of gene and protein structure, but we added annotation for the part of the protein coded by exon 2 in MEC-3a. According to the Pfam annotation on Wormbase, there are two LIM domains at 29-83 a.a. and 89-146 a.a. Exon 2 codes the amino acids 27-43 in the MEC-3a isoform. So, the absence of exon 2 in MEC-3c and MEC-3d indeed will truncate the first LIM domain.**

6. To improve clarity, the authors can show locations of the primers they used in figures illustrating gene loci/exon structures. For example in Fig3B for gene egl-8 it’s unclear where the primers sit on the gene structure. Is the first exon, exon1? Which exon is the last one represented? This could be made clearer.

***Response*: We thank the reviewer for the great suggestion. To indicate the location of the primers in the gene structure, we added the exon number of the first exon shown in the first isoform of each gene in Figure 3B. For example, based on Wormbase gene annotation, the primer for *egl-8* binds to the junction between exon 8 and 9 of isoform a, so we labeled exon 8 in the Figure panel, which serves as the landmark for the location of other primers for *egl-8*.**

Line-by-line comments:

Line16 – regulates

***Response*: Corrected**

Line22 - dash missing in MLB-1

***Response:* We did not find this typo.**

Line25 - is instead of are

***Response:* Corrected**

Figure 1F - Very hard to tell the difference in GFP+RFP between the two channels. Would be better to split and add merge like FigS1B

***Response:* The purpose is to show that *mbl-1p::GFP* reporter overlaps with the TRN-specific RFP reporter. We think it is quite easy to see this overlapping with the merge images alone. We added some arrows to make it clearer.**

Line137 - dash missing in mbl-1

***Response:* We did not find this typo.**

Fig 2B - need to add Y label title to make it clearer

***Response:* We added the Y axis label in the revised Figure 2.**

Line170 - It could helpful (especially to people outside the field) if the authors add one sentence explaining why they are using mitochondria as an example of MT-mediated cargo transport

***Response:* We thank the reviewer for the suggestion and added a sentence “In neurons, mitochondria are transported to the axons in a MT-dependent manner.”**

Line206 - Authors don’t know for sure that MBL-1 function is unique. A more accurate statement would be “ability to control neural growth not general to all splicing factors”

***Response:* We agree with the reviewer and changed the sentence to “the ability of MBL-1 to regulate neurite growth is not shared by the other two splicing factors.”**

Line237 - The OE of ptl-1 isoforms and the effect in ALM-PN seems to miss from FigS3

***Response:* The results are now added to Figure S3.**

Line303 - typo in “/-tubulin” ?

***Response:* We did not find this typo. It could be that the symbol “α” or “β” did not show up in the version reviewer received.**

Line306 - Fig4D does not show results for mbl-1 (-) allele

***Response:* Figure 4D shows examples for *mec-3(a)* and *mec-3(d)* alleles. For *mec-17p::TagRFP* expression in *mbl-1(-)* mutants, the quantification is shown in Figure 4G. We added representative images in Figure S4F.**

Line 384 - missing dash in mbl-1

***Response:* We did not find this typo.**

Line 418 - Typo “loss-offunction”

***Response:* We did not find this typo.**

Line446-451- This paragraph could be misleading and should be changed. The fact that the authors did not find MAPK pathway genes as candidates in their RNAseq is not sufficient evidence to suggest mbl-1 does not regulate genes in this pathway.

***Response:* We toned down the sentence to “…, suggesting that they were may not be the direct targets of MBL-1. Instead, MBL-1 may indirectly control the activity of the pathway.” Since MBL-1 is an RNA-binding protein and a splicing regulator, if the RNA level and splicing pattern of a gene is not affected by MBL-1, we assume that the gene is not a direct target of MBL-1. But we could not rule out the possibility that MBL-1 may indirectly control the activity of this pathway through other proteins.**
